# Authors’ Response to Peer Reviews of “AI-Driven and Automated Systems for Continuous Oxygen Saturation Monitoring in Long-Term Oxygen Therapy: Systematic Review”

**DOI:** 10.2196/111441

**Published:** 2026-10-07

**Authors:** Suman Kadariya, Prajita Niraula, Bishal Poudel, Sujan Kadariya

**Affiliations:** 1 Conway Regional Medical Center, Conway, AR, United States; 2 Independent Researcher, Redcross Rd, Kathmandu, 44600, Nepal, 977 9818787009; 3 KIST Medical College, Kathmandu, Nepal; 4 Kathmandu University School of Medical Sciences, Dhulikhel, Nepal

**Keywords:** long-term oxygen therapy, artificial intelligence, machine learning, oxygen saturation monitoring, pulse oximetry, closed-loop systems, automated oxygen titration, motion artifacts, chronic obstructive pulmonary disease, systematic review

*This is the authors’ response to peer-review reports for “AI-Driven and Automated Systems for Continuous Oxygen Saturation Monitoring in Long-Term Oxygen Therapy: Systematic Review.”*

## Round 1 Review

### Reviewer AA [1]

#### Specific Comments

##### Major Comments

This paper [2] aims to evaluate AI-based and automated systems for long-term oxygen therapy (LTOT), addressing a timely and clinically relevant topic given the rapid advancement in personalized and responsive respiratory care technologies. The systematic approach and effort to integrate performance and usability dimensions are commendable. However, in its current form, the manuscript requires substantial revisions before it can be considered for publication. In particular, I would like to highlight the following concerns.

1. Lack of clearly defined research goal and review question.

The research goal and review question are not clearly described. This makes it difficult to understand whether the review is meant to summarize existing technologies, compare the performance of AI and automated systems, or evaluate how these systems can be applied in real clinical settings.

**Response:** We added 3 explicit research questions and organized the manuscript around technical performance, clinical performance, and equity/home deployment readiness.

**Manuscript change:** Research questions added at the end of the Introduction and reflected in the Results/Discussion.

2. Insufficient background on technological context and rationale.

In the Introduction section, the paper should provide more background to help readers understand why the new approach is important. It should briefly explain how LTOT technology has changed over time, what makes AI-based systems different, and why this review is timely and needed. While the Introduction mentions some clinical problems that AI aims to solve—such as inefficiency of fixed flow and poor response to activity changes—it does not explain how older technologies tried to handle these issues or where they fell short.

**Response:** We expanded the Introduction with a technology-history narrative and specific AI/automation categories.

**Manuscript change:** Background strengthened and linked to review rationale.

3. Misalignment between stated objectives and inclusion criteria.

Although the Introduction emphasizes the need for a comprehensive review of AI technologies in the context of LTOT, the actual inclusion criteria are limited to studies addressing technical challenges such as motion-induced signal distortions (eg, convolutional neural network–based denoising, wavelet transforms), low-perfusion signal management, mitigation of skin tone–related measurement bias, and signal stability over 24 hours.

**Response:** We reframed the scope around LTOT-critical technical and clinical prerequisites and explained why motion artifact, low perfusion, skin tone bias, and signal stability are clinically relevant.

**Manuscript change:** Eligibility criteria now include the rationale for each technical criterion.

4. Unclear populations in reviewed studies.

The manuscript does not clearly describe which patient populations were included in the evaluation of AI or automated systems. Without this information, it is difficult to assess the clinical relevance and generalizability of the findings. Given that LTOT is most commonly used in older adult or chronic obstructive pulmonary disease populations, it is essential to specify whether the reviewed studies included such groups, especially in real-world or home-care settings.

**Response:** We expanded Table 1 and the Results narrative to summarize patient/volunteer/dataset populations, chronic obstructive pulmonary disease relevance, and gaps in demographic reporting.

**Manuscript change:** Population and setting details were added to Table 1 and the Results section.

##### Minor Comments

1. Please consider expanding the summary table to include more descriptive information (for example, publication year and country, participant characteristics, and study design). Also, to reduce confusion, organize key system outputs such as SpO_₂_ accuracy, motion artifact handling, and skin tone bias under a single “Outcomes” category rather than listing them individually.

**Response:** We expanded Table 1 with these fields and consolidated outcomes into Primary Outcomes and Key Metrics.

**Manuscript change:** Table 1 revised.

2. Abbreviations should be written out in full only once when first introduced. There is no need to repeat the full terms (abbreviations) in later sections such as the Discussion or Future Directions section.

**Response:** We reduced redundant abbreviation expansion and retained an alphabetized Abbreviations section.

**Manuscript change:** Abbreviations cleaned and listed before the References.

### Reviewer CR [3]

#### General Comments

The authors conducted a systematic review on LTOT. While the topic is relevant and potentially valuable, the manuscript in its current form is poorly formatted and difficult to follow. Revision is needed in terms of structure, clarity, and presentation.

#### Specific Comments

##### Major Comments

1. The paper does not clearly summarize the key research questions it aims to address, and the answers presented throughout the manuscript are not well organized. A concise paragraph at the end of the Introduction would help readers understand the scope, objectives, and overall structure of the paper before proceeding to the main sections. In addition, the boundaries between the Methods, Results, and Discussion sections are unclear, with content from these sections often mixed together.

**Response:** We added an orientation paragraph and reorganized the Methods, Results, and Discussion so methods, findings, and interpretation are separated.

**Manuscript change:** IMRD boundaries clarified.

2. In the Methods section, the subsections “Literature Search and Article Selection” and “Study Selection and Eligibility Criteria” appear in an illogical order, and parts of the article selection process are repeated across both subsections. Similar organizational issues appear in the Results section with “Overview of Included Studies” and “Study Characteristics,” where content overlaps substantially. Furthermore, in the “Overview” section, the manuscript mainly describes included studies one by one, which limits synthesis and makes it harder for readers to identify broader patterns across the literature.

**Response:** We reordered the Methods section in the PRISMA (Preferred Reporting Items for Systematic Reviews and Meta-Analyses) sequence and merged overlapping Results section overview/characteristics material into a concise synthesis plus Table 1.

**Manuscript change:** The Methods and Results were restructured.

3. The manuscript also contains several broad claims that are not adequately supported by evidence. For example, statements such as “few studies discussed failure cases, outliers, or conditions under which the system underperformed” and “many used publicly available datasets (eg, PhysioNet) that lacked full clinical context or were limited in demographic diversity” should be supported by specific citations or a clearer summary of which studies these statements refer to.

**Response:** We revised broad claims to specify which studies did or did not report relevant findings, especially failure modes, dataset limitations, and demographic stratification.

**Manuscript change:** Study-specific citations added throughout the Results and Discussion sections.

4. Separating methodologies into AI and non-AI categories seems somewhat arbitrary, particularly when the review is not explicitly framed around AI in LTOT. The rationale for this categorization should be clarified or reconsidered.

**Response:** We added operational definitions and explained why learning-based AI and deterministic closed-loop automation have different evidence gaps and clinical roles.

**Manuscript change:** AI vs non-AI classification rationale added.

##### Minor Comments

5. The manuscript also requires a careful check of formatting consistency. Citation style is currently inconsistent, with some references presented as numbers, while others use the author-year format. For example, the sentence “The models used techniques such as deep neural networks and Gaussian process regression, often trained on datasets curated under controlled environments (Argüello-Prada & Castillo, 2024)” does not match the numbered citation style used elsewhere in the paper. In addition, subsection formatting is inconsistent, as some subtitles are numbered, while others are not. These issues should be revised to improve professionalism and readability.

**Response:** We standardized citations, removed author-year citations, normalized section headings, and removed unfinished appendix material from the main manuscript.

**Manuscript change:** Formatting and citation consistency improved.

We again thank the editor and reviewers for strengthening the manuscript. The revised manuscript and accompanying files have been prepared to improve transparency, reproducibility, and alignment with JMIRx Med requirements.

## Abbreviations

LTOT: long-term oxygen therapy
